# Outcome Determinants of Stroke in a Brazilian Primary Stroke Center

**DOI:** 10.1155/2014/194768

**Published:** 2014-12-11

**Authors:** Gustavo W. Kuster, Lívia A. Dutra, Israel P. Brasil, Evelyn P. Pacheco, Márcio A. C. Arruda, Cristiane Volcov, Renan B. Domingues

**Affiliations:** ^1^Hospital Paulistano, 01321-001 São Paulo, SP, Brazil; ^2^Stroke Integrated Program (PIAVEN), Amil Stroke Network, 01321-001 São Paulo, SP, Brazil; ^3^Department of Neurology, Faculdade de Medicina do ABC, 09060-650 Santo André, SP, Brazil; ^4^Department of Neurology, Federal University of São Paulo (UNIFESP), 04021-001 São Paulo, SP, Brazil; ^5^Neurosciences Program, Federal University of Minas Gerais (UFMG), 31270-901 Belo Horizonte, MG, Brazil

## Abstract

*Background.* Stroke mortality in Brazil is one of the highest among Western countries. Nonetheless, stroke outcome determinants are still poorly known in this country. In this study we evaluate outcome determinants of stroke in a primary stroke center in São Paulo, Brazil.* Methods.* We evaluated demographic, clinical, and outcome data of patients with ischemic stroke (IS), transient ischemic attack (TIA), and intracerebral hemorrhage (ICH) admitted at “Hospital Paulistano,” São Paulo, Brazil. In-hospital mortality and functional outcome determinants were assessed. Univariate and binary logistic regression analysis were performed.* Results.* Three hundred forty-one patients were included in the study, 52.2% being male with 66.8 ± 15.7 years. The stroke type distribution was IS: 59.2%, TIA: 29.6%, and ICH: 11.1%. ICH was associated with greater severity and poorer functional outcome. The determinants of poorer functional outcome were higher NIHSS, lower Glasgow score, and lower oxygen saturation level. The most important mortality determinant was the presence of visual symptoms.* Conclusions.* The stroke mortality and stroke outcome determinants found in the present study do not remarkably differ from studies carried out in developed countries. Stroke prognosis studies are crucial to better understand the high burden of stroke in Brazil.

## 1. Introduction

Stroke is the first cause of disability, the second cause of cognitive impairment, and the third cause of death in the world [1]. Two-thirds of all stroke deaths occur in low- and middle-income countries, including Latin America countries; however, stroke has been poorly studied in these regions [2]. In Brazil, the largest country in the region, stroke is the leading cause of death and disability and the stroke mortality is one of the highest among Western countries [3, 4].

The causes of the high stroke burden in Brazil remain speculative. One possibility is that the prevalence of stroke is higher in Brazil than in other regions. Some prevalence studies were carried in Brazil but the data are not sufficient to provide an overview of stroke prevalence in Brazil, since most of these studies were carried out in specific regions that do not necessarily reflect the whole country reality [5]. Other potential explanations rely on medical assistance deficiencies, mostly driven by socioeconomic inequality. In fact, previous studies showed that the majority of patients are assisted in public hospitals where diagnostic and therapeutic resources are scarce [6–9]. However, the negative impact of these discrepancies on stroke outcome in Brazil still requires confirmation by large population studies. At this point few studies have evaluated stroke outcome [7–10] in Brazil and the outcome determinants of stroke in Brazil are still poorly known.

Several variables including the higher score with NIH Stroke Scale (NIHSS), higher age, atrial fibrillation, hypertension, diabetes mellitus, lower level of consciousness, higher time between stroke onset and admission, and the presence of visual field abnormalities at the first evaluation have been considered predictors of stroke poor outcome [11–16]. Most of the outcome determinants studies were carried out in developed or Asian countries.

The aim of the present study was to evaluate stroke prognosis and stroke prognosis determinants in a population of stroke patients seen in a primary stroke center in São Paulo, the largest Brazilian city.

## 2. Methods

This study was carried out between 2012 and 2014 at Hospital Paulistano, a Joint Commission International certified stroke center located in the central region of São Paulo, Brazil. This study protocol was approved by the Ethics Committee on Research of Hospital Paulistano (Amil Stroke Network) and informed consent was obtained from each participant or legal representative.

### 2.1. Patients and Procedures

Patients that presented to the emergency room with suspected stroke were evaluated by a neurologist at admission and submitted to a standardized protocol. Demographical data, time delay between stroke onset and admission, stroke warning symptoms (weakness and numbness, confusion, speech difficulties, comprehension problems, vision abnormalities, walking troubles, severe headache, dizziness, and incoordination), and stroke risk factors (hypertension, smoking, contraceptive use, obesity, familial history, diabetes, previous stroke, or transient ischemic attack) were compiled. We also collected the following data at admission: vital signs, capillary glucose, oxygen saturation levels, Glasgow coma scale score, and National Institutes of Health Stroke Scale (NIHSS).

After initial evaluation patients were submitted to noncontrast brain CT with 10-mm slice thickness and radiological findings (detectable brain infarct, dense artery sign, local intracerebral hemorrhage, distant brain hemorrhage, and cerebral edema) were recorded. Patients were diagnosed as having ischemic stroke (IS), transient ischemic attack (TIA), intracerebral hemorrhage (ICH), or subarachnoid hemorrhage (SHA) according to previously established definitions [17]. Data of IS treatment including thrombolytic therapy, the use of antithrombotic agents in the first 48 hours, and venous thrombosis prophylaxis were collected. Patients without confirmed stroke and patients with SHA were not included. The outcome determinants analysis was carried exclusively in the groups of patients with confirmed IS or ICH.

### 2.2. Statistical Analysis

Statistical analysis was performed using SPSS version 15.0 for Windows. The confidence interval was of 95% and the significance level was set at *P* < 0.05. Normality was assessed using the Kolmogorov-Smirnov test. Student's *t*-test was used for continuous data and categorical data were compared using Chi-square analyses. In-hospital mortality and modified Rankin score (mRs) at discharge, whereby the patients were divided into two groups, one with mRs ≤ 2 and other with mRs > 2, were defined as outcomes measures. Binary logistic regression analysis was performed after excluding nonsignificant variables with in-hospital mortality and discharge Rankin modified score as dependent variables.

## 3. Results

Demographic data, stroke risk factors, stroke warning symptoms, and clinical data from the 341 patients included in the study are shown in Table 1.

The therapeutic measures adopted in patients with brain ischemia were as follows: twenty-seven out of the 202 patients with IS (13.4% of all IS patients) were treated with thrombolytic treatment, 18 (8.9%) with intravenous and 9 (4.4%) with endovascular therapy. Two hundred fifty-eight out of 303 patients with IS and TIA (85.1%) received antithrombotic therapy within 48 hours after stroke and 93.1% of the group of patients with either IS or TIA received venous thrombosis prophylactic treatment. Among ICH patients, nine (23.7%) were submitted to surgical treatment and the others received conservative treatment.

When comparing the clinical features and outcome of patients with IS and ICH, we found that patients with ICH had a more severe disease than patients with IS. Patients with IS had higher diastolic blood pressure (IS: 82 ± 15.6, ICH: 100.9 ± 120, *P* = 0.011), lower oxygen saturation levels (IS: 96 ± 2.7, ICH: 94.9 ± 4.5, *P* = 0.038), and lower Glasgow score at admission (IS: 14.3 ± 1.9, ICH: 12.5 ± 3.8, *P* < 0.001). At discharge patients with ICH had higher NIHSS scores (IS median = 0, interquartile range = 2, ICH median = 2.5, interquartile range = 10, and *P* < 0.001) and higher mRS (IS median = 0, interquartile range = 2, ICH median = 1.5, interquartile range = 4, and *P* < 0.001).

The outcome measures were evaluated in patients with IS and ICH. Twenty-eight out of 315 patients (8.9%) died during hospitalization, 23 (8.2%) of the patients with IS and 5 (13.9%) of the patients with ICH (*P* = 0.256). At hospital discharge 31.7% of the survivors had mRs > 2, 28.1% of the IS patients and 62.5% of ICH patients (*P* < 0.001). Tables 2 and 3 show the comparisons of characteristics of patients according to the outcome measures. After binary regression analysis the only variables significantly associated with mRs > 2 at discharge were ICH diagnosis (*P* < 0.001), lower oxygen saturation (*P* = 0.026), lower Glasgow score (*P* = 0.008), and higher NIHSS at onset (*P* < 0.001). The only variable associated with in-hospital death was the presence of vision field abnormality at admission (*P* = 0.018).

## 4. Discussion

To our knowledge this was the first study to evaluate outcome determinants in a Brazilian primary stroke center. We found that lower Glasgow score, lower NIHSS, and lower oxygen saturation were independently associated with poorer functional outcome and that visual symptoms at onset were independently associated with in-hospital mortality. These findings are in line with previous studies. NIHSS has been demonstrated as one of the most important outcome predictors among patients with IS [13–16]. Reduced level of consciousness and reduced Glasgow score were found to be associated with poorer outcome in patients with IS [16] and in patients with ICH [18, 19]. Lower oxygen saturation at admission is inversely correlated with stroke prognosis [20]. Visual field abnormalities were associated with unfavorable outcome in patients with IS in a previous study [16]. The association of visual symptoms with poorer outcome could be related with posterior circulation involvement, explaining the worse outcome. Other variables were previously associated with poorer outcome: age, hypertension, diabetes and glucose level at admission, CT hyperdense artery sign, and CT hypointensity [16, 21, 22]. In our study some variables were associated with outcome only by univariate analysis. Patients with poorer functional outcome were of higher age and had higher glucose level, more frequent confusion, vision abnormalities, CT hypodensity, and cerebral edema; however, these differences were not significant after adjusted analysis. CT dense artery sign and cerebral edema were more frequent among those who died during hospitalization; however, these differences were no longer significant after regression analysis. It is possible that the small sample size in the present study did not allow the demonstration of other significant differences.

The mean stroke age we found was similar to what was found in a recent study in United States [23]. ICH proportion and ICH severity were similar to previous descriptions [24]. The therapeutic measures registered here are in accordance with stroke care quality indicators. The percentage of IS patients treated with tPA in our study was even higher than in previous population studies [25], probably reflecting the fact that our study was carried in a single institution with 24-hour neurologist and a stroke protocol. The rate of patients that received antithrombotic therapy within the first 48 hours after stroke (85.1%) and the rate of patients submitted to venous thrombosis prophylactic treatment (93.1%) are similar to what was previously reported in US hospitals [26, 27] and in other private hospitals in Brazil [28]. The in-hospital mortality rate registered here was not discrepant from previous studies in developed countries [29]. Overall these data suggest that the clinical, therapeutic, and outcome characteristics of the population of the present study do not remarkably differ from stroke populations in developed countries. It is likely that this reflects the fact that the present study was carried out in a private institution, with availability of all stroke validated therapies. Our hospital serves predominantly middle and upper middle class patients. The emergence service of our hospital has a neurologist 24 hours a day, 7 days a week, a nurse in charge of the management of stroke cases, intensive care unit, 24-hour CT scan, magnetic resonance imaging, and an interventional neuroradiology service available 24 hours a day, 7 days a week. It is a primary stroke center certified by Joint Commission International that follows the American Heart Association guidelines. Considering the specificities of our service, large and multicentric studies are still needed to determine if the outcome of stroke among patients assisted in public Brazilian hospitals differs from private hospitals.

Our study has some limitations that deserve to be mentioned. We analyzed a small and a single center sample. Our results need confirmation in larger and multicentric studies involving different Brazilian regions with different socioeconomic levels. Another limitation is that this study did not include follow-up of the patients and we evaluated only in-hospital outcomes, while most of the prognostic studies assess three-month outcome. Future outcome studies should address long-term outcome in patients with stroke in Brazil. As strengths of this study the population was homogeneous and the same stroke team assisted all the patients following the same stroke protocols.

In conclusion, the present study was the first to specifically assess outcome determinants in a Brazilian primary stroke center with availability of all current validated stroke therapies. The outcome and outcome determinants reported in the present study shared great similarities with previous studies in developed countries. Population studies are still necessary to better understand the great burden of stroke in Brazil.

## Figures and Tables

**Table 1:** Demographical, clinical, and radiological data of the 341 patients included in the study.

Characteristics	Findings
Age (mean ± SD)	66.8 ± 15.7 years
Gender	
Male (%)	52.2
Female (%)	47.8
Type of stroke	
IS (%)	59.2
TIA (%)	29.6
ICH (%)	11.1
Previous hypertension (%)	68
Diabetes (%)	37
Previous stroke (%)	26.4
Smoking (%)	14
Obesity (%)	7.9
Familial history of stroke (%)	7
Time of symptoms at arrival^*^ (mean ± SD)	1661 ± 3979 minutes
Wake-up stroke (%)^*^	25.2
Stroke warning symptoms^*^	
Weakness and numbness (%)	66.6
Confusion, speech difficulties, and comprehension problems (%)	39
Dizziness and incoordination (%)	19.6
Severe headache (%)	16.7
Walking troubles (%)	16.4
Vision abnormalities (%)	11.1
Systolic blood pressure (mean ± SD)^*^	148.9 ± 54.1 mm Hg
Diastolic blood pressure (mean ± SD)^*^	84.1 ± 42.8 mm Hg
Heart rate (mean ± SD)^*^	80.8 ± 15.9 beats per minute
Oxygen saturation level (mean ± SD)^*^	95.9 ± 3%
Capillary glycemia (mean ± SD)^*^	137.1 ± 56.1 mg/dL
Temperature (mean ± SD)^*^	35.9 ± 2.9 Celsius
Glasgow score (mean ± SD)^*^	14.1 ± 2.3
NIHSS at admission^*^	Median = 2, interquartile range = 5
NIHSS at discharge^**^	Median = 0, interquartile range = 2

^*^Data obtained at the first evaluation; ^**^data obtained at discharge; IS: ischemic stroke; ICH: intracerebral hemorrhage; TIA: transient ischemic attack; NIHSS: National Institutes of Health Stroke Scale.

**Table 2:** Comparison of clinical and radiological data according to the modified Rankin score (mRs) at discharge.

	mRs ≤ 2 (*N* = 157)	mRs > 2 (*N* = 73)	*P*	*P* ^*^
Age	65.1 ± 15.5	72.2 ± 15	**<0.001**	0.398
Male	90 (39.1%)	36 (15.7%)	0.256	—
Smoking	25 (10.1%)	9 (3.9%)	0.463	—
Hypertension	105 (45.7%)	58 (25.2%)	0.061	—
Obesity	15 (6.5%)	7 (3%)	0.993	—
Family history	12 (5.2%)	4 (1.7%)	0.548	—
Diabetes	60 (26.1%)	29 (12.6%)	0.827	—
Previous stroke	42 (18.3%)	19 (8.3%)	0.908	—
Weakness and numbness	109 (47.4%)	54 (23.5%)	0.307	—
Confusion, speech difficulties, and comprehension problems	53 (23%)	38 (16.5%)	**0.017**	0.130
Vision abnormalities	24 (10.4%)	2 (0.9%)	**0.012**	0.998
Walking troubles	22 (9.5%)	18 (7.8%)	0.071	—
Severe headache	27 (11.7%)	7 (3%)	0.147	—
Dizziness and incoordination	33 (14.3%)	11 (4.8%)	0.239	—
CT detectable brain infarct	25 (10.9%)	20 (8.7%)	**0.035**	0.778
CT dense artery sign	3 (1.3%)	7 (3%)	0.073	—
Cerebral edema	4 (1.7%)	11 (4.8%)	**0.004**	1.000
Wake-up stroke	35 (15.2%)	26 (11.3%)	0.068	—
SBP	147.3 ± 25.8	147.2 ± 29.5	0.973	—
DBP	86.8 ± 60.4	84.5 ± 15	0.745	—
HR	80.4 ± 15.5	81.8 ± 19.3	0.563	—
Sat O2	96.5 ± 2.1	94.4 ± 4.5	**<0.001**	**0.026**
Temperature	35.9 ± 2.9	35.6 ± 4.4	0.555	—
Digital glycemia	130.8 ± 50.1	148.7 ± 62	**0.040**	0.846
Time of symptoms	1359.6 ± 2697	1850.8 ± 4091.4	0.330	—
Glasgow (initial)	14.7 ± 1.4	13.1 ± 3.2	**<0.001**	**0.008**
NIHSS (initial)	Median = 1, IR = 3	Median = 12, IR = 14	**<0.001**	**<0.001**

CT: computed tomography; SBP: systolic blood pressure; DBP: diastolic blood pressure; HR: heart rate; Sat O2: oxygen saturation; NIHSS: National Institutes of Health Stroke Scale; IR = interquartile range; *P*: according to *t*-test (continuous variables) or Chi-square test (categorical variables); *P* ^*^: according to binary logistic regression analysis with significant variables.

**Table 3:** Comparison of clinical and radiological data of survivors and patients that died during hospitalization.

	Survivors (*N* = 287)	In-hospital death (*N* = 28)	*P*	*P* ^*^
Age	66.4 ± 15.8	68.5 ± 16.7	0.509	—
Male	156 (49.5%)	15 (4.8%)	0.555	—
Smoking	44 (14%)	4 (1.3%)	0.904	—
Hypertension	194 (61.6%)	22 (7%)	0.361	—
Obesity	23 (7.3%)	4 (12.7%)	0.498	—
Family history	20 (6.3%)	2 (0.6%)	0.001	—
Diabetes	107 (34%)	9 (2.8%)	0.652	—
Previous stroke	75 (23.8%)	9 (2.8%)	0.189	—
Weakness and numbness	192 (60.9%)	19 (6%)	0.179	—
Confusion, speech difficulties, and comprehension problems	111 (35.2%)	10 (3.2%)	0.121	—
Vision abnormalities	**27 (8.6%)**	**7 (2.2%)**	**0.024**	**0.018**
Walking troubles	47 (14.9%)	5 (1.6%)	0.072	—
Severe headache	48 (15.2%)	7 (2.2%)	0.135	—
Dizziness and incoordination	60 (19%)	5 (15.9%)	0.064	—
CT detectable brain infarct	56 (17.8%)	7 (2.2%)	0.538	—
CT dense artery sign	13 (4.1%)	3 (0.9%)	**0.005**	0.599
Cerebral edema	18 (5.7%)	2 (0.6%)	**0.002**	1.000
Wake-up stroke	78 (24.8%)	4 (1.3%)	0.282	—
SBP	150.2 ± 58	142.1 ± 24.7	0.165	—
DBP	84.8 ± 46.2	81.8 ± 15.6	0.463	—
HR	80.7 ± 15.8	78.2 ± 19.5	0.516	—
Sat O2	95.8 ± 3.2	96.5 ± 2.4	0.180	—
Temperature	35.9 ± 3.1	36 ± 0.4	0.460	—
Digital glycemia	136.6 ± 55	122 ± 33.9	0.100	—
Time of symptoms	1777.7 ± 4215.2	773.25 ± 1180.7	0.009	0.252
Glasgow (initial)	5.4 ± 7.5	6 ± 7.6	0.685	0.293
NIHSS (initial)	Median = 2, IR = 8	Median = 2, IR = 10	0.694	0.125

CT: computed tomography; SBP: systolic blood pressure; DBP: diastolic blood pressure; HR: heart rate; Sat O2: oxygen saturation; NIHSS: National Institutes of Health Stroke Scale; IR = interquartile range; *P*: according to *t*-test (continuous variables) or Chi-square test (categorical variables); *P* ^*^: according to binary logistic regression analysis with significant variables.
